# Fungal Biofilms as a Valuable Target for the Discovery of Natural Products That Cope with the Resistance of Medically Important Fungi—Latest Findings

**DOI:** 10.3390/antibiotics10091053

**Published:** 2021-08-30

**Authors:** Estefanía Butassi, Laura Svetaz, María Cecilia Carpinella, Thomas Efferth, Susana Zacchino

**Affiliations:** 1 Pharmacognosy Area, School of Biochemical and Pharmaceutical Sciences, Universidad Nacional de Rosario, Suipacha 531, Rosario 2000, Argentina; fefabutassi@hotmail.com (E.B.); laurasvetaz@hotmail.com (L.S.); 2 Fine Chemical and Natural Products Laboratory, IRNASUS CONICET-UCC, Universidad Católica de Córdoba, Córdoba 5016, Argentina; ceciliacarpinella@ucc.edu.ar; 3 Institute of Pharmaceutical and Biomedical Sciences, Johannes Gutenberg University, Staudinger Weg 5, 55128 Mainz, Germany; efferth@uni-mainz.de

**Keywords:** fungal biofilm, antifungal resistance, natural products, *Candida* spp., *Fusarium* spp., *Cryptococcus* spp., filamentous fungi, mechanisms of antibiofilm action

## Abstract

The development of new antifungal agents that target biofilms is an urgent need. Natural products, mainly from the plant kingdom, represent an invaluable source of these entities. The present review provides an update (2017–May 2021) on the available information on essential oils, propolis, extracts from plants, algae, lichens and microorganisms, compounds from different natural sources and nanosystems containing natural products with the capacity to in vitro or in vivo modulate fungal biofilms. The search yielded 42 articles; seven involved essential oils, two Brazilian propolis, six plant extracts and one of each, extracts from lichens and algae/cyanobacteria. Twenty articles deal with the antibiofilm effect of pure natural compounds, with 10 of them including studies of the mechanism of action and five dealing with natural compounds included in nanosystems. Thirty-seven manuscripts evaluated *Candida* spp. biofilms and two tested *Fusarium* and *Cryptococcus* spp. Only one manuscript involved *Aspergillus fumigatus*. From the data presented here, it is clear that the search of natural products with activity against fungal biofilms has been a highly active area of research in recent years. However, it also reveals the necessity of deepening the studies by (i) evaluating the effect of natural products on biofilms formed by the newly emerged and worrisome health-care associated fungi, *C. auris*, as well as on other non-*albicans Candida* spp., *Cryptococcus* sp. and filamentous fungi; (ii) elucidating the mechanisms of action of the most active natural products; (iii) increasing the in vivo testing.

## 1. Introduction

In recent decades, fungi has emerged as a major cause of life-threatening invasive human infections, in particular among immunocompromised patients [1,2,3], particularly those with human immunodeficiency virus (HIV), cancer patients receiving chemotherapy, transplant recipients, extremely aged persons and subjects in intensive care units [4,5]. Fungal infections lead to mortalities estimated in 1.5 million per year, having a great impact on global human health [5]. The main purpose of this review is to provide an updated analysis of the natural products with a capacity of inhibiting fungal biofilms, published from 2017 to May 2021.

We collected papers on essential oils (EOs), propolis, extracts from plants, algae, lichens and microorganisms, metabolites obtained from these sources and nanosystems.

### 1.1. Most Common Etiological Agents Causing Fungal Infections

The most common fungi identified in systemic infections are the yeasts of the *Candida* and *Cryptococcus* genera, as well as the filamentous fungi of the *Aspergillus* genus [6,7,8]. *Candida* spp. produce a 40% mortality rate [2,9], while cryptococcosis and aspergillosis produce a mortality rate between 20% to 90% [9]. Among *Candida* spp., *C*. *albicans* showed to be the fourth most common cause of nosocomial bloodstream infections [10], although a recent global shift in epidemiology towards non-*albicans Candida* spp., such as *C*. *glabrata*, *C*. *parapsilopsis*, *C*. *tropicalis*, *C*. *krusei* and lastly, *C*. *auris,* has been detected [11,12]. Apart from the mentioned fungi, other species such as *Pneumocystis jirovecii*, *Histoplasma capsulatum* and the mucormycetes of *Mucor*, *Absidia* and *Rhizopus* genera, are important fungal pathogens responsible for the majority of serious fungal diseases [13,14,15].

### 1.2. Available Antifungal Agents

Currently, five classes of antifungal agents, the polyenes, azoles, allylamines, echinocandins and pyrimidines are used for the treatment of fungal infections in human beings [16,17]. Some of them target ergosterol, an essential component of the fungal membrane, either by binding to it (i.e., polyenes such as amphotericin B) or by interfering with different steps of its biosynthesis (i.e., triazoles and allylamines) [16,17]. In particular, the interaction or sequestration of ergosterol by amphotericin B disturbs the membrane, which leads to an increased permeability and the leakage of the intracellular components resulting in the death of the pathogen [18,19,20]. Previous publications demonstrated that amphotericin B is also able to induce the generation of reactive oxygen species (ROS), as an additional mechanism to achieve the fungicidal activity [21]. On the other hand, the triazoles (i.e., fluconazole, voriconazole, itraconazole, isavuconazole and posaconazole) inhibit the enzyme lanosterol 14α-demethylase responsible for the demethylation of 14α-lanosterol [16] to form ergosterol in a process dependent on the cytochrome P450 system [22,23]. Allylamines, such as terbinafine, interfere with the activity of squalene epoxidase, the enzyme that catalyzes the stereospecific epoxidation of squalene to 2,3-(*S*)-oxidosqualene involved in ergosterol synthesis [24,25,26]. In addition to ergosterol, 1,3-β glucan, present in fungal cell walls, is another target for antifungal drugs due to its role in fungal growth, integrity and division as well as its participation as a virulence factor [27,28]. The echinocandins (i.e., caspofungin, micafungin and anidulafungin) exhibit a noncompetitive inhibition of the 1,3-β-d-glucan synthase, leading to a deficiency of this polymer [23,29]. The pyrimidine analogue flucytosine, selectively interferes with fungal DNA synthesis by inhibiting the thymidylate synthetase, a key enzyme acting as a crucial source of thymidine. 

Although the different types of discovered antifungal drugs have enabled progress in the management of fungal infections, serious problems regarding side effects, limited options of antifungals and mainly the development of drug resistance [30,31,32,33], highlight the urgent need of exploring new therapeutic approaches that overpass the unmet clinical needs [34,35,36]. 

### 1.3. Fungal Biofilms 

**Figure 1:**
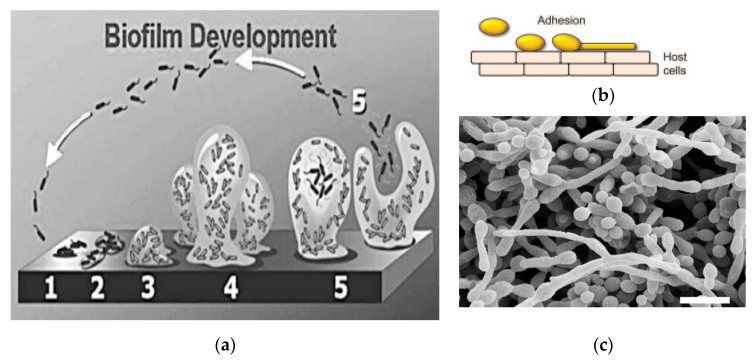
(**a**) The five-stage process involved in the development of biofilm: 1. adherence of yeasts to a surface followed by yeast-to-hyphal transition; 2. the exopolymeric matrix (EPM) is produced resulting in a firmly adhered “irreversible” attachment; 3. early biofilm architecture is developed; 4. the biofilm reaches maturation in a three-dimensional structure and 5. single planktonic cells are dispersed from the mature biofilm. Reproduced from Stoodley et al., 2002 [49]. Image credit: D. Davies, with permission of Prof. David Davies. (**b**) A clearer scheme of stage 1. Yeasts adhere to host cell surfaces. Contact to host cells triggers the yeast-to-hyphal transition and directed growth via thigmotropism. Reproduced from part of Figure 1a from Mayer et al. [51], with permission. (**c**) A scan electron microscopy (SEM) image of a mature (48 h) *Candida albicans* biofilm (formed in stage 4 of Figure 1a). Bar = 10 µm. Yeasts, hyphae, and pseudohyphae can be observed. Most EPM was lost during the SEM procedures. Reproduced from Ramage et al. [50] with permission.

The most difficult-to-eradicate mycoses are not caused by planktonic cells, but by immobilized fungi (sessile cells) that form well-structured biofilms with the ability to adhere to different surfaces, organs or to medical devices such as catheters [37,38]. Biofilms (Figure 1) are the predominant growth lifestyle of many opportunistic fungal pathogens, e.g., *albicans* and non-*albicans Candida* spp., *Cryptococcus neoformans*, *Cryptococcus gatti*, *Trichosporon asahii*, *Rhodotorula* spp., *Aspergillus fumigatus*, *Malassezia pachydermatis*, *Histoplasma capsulatum*, *Coccidioides immitis*, *Pneumocystis* spp., *Fusarium* spp. and many others [39]. The National Institute of Health estimated that microbial biofilms were responsible for over 80% of all infections [40].

A biofilm is defined as a community of microorganisms encapsulated in a self-produced exopolysaccharide matrix (EPM) attached to a biotic or abiotic surface [41,42], which plays a key role in its resistance [43]. The composition of the EPM produced by different fungi has been reviewed by Mitchell et al. [44]. Particularly, it contains carbohydrates, hexosamine, phosphorus, proteins, uronic acid and environmental DNA (eDNA) which were initially identified in *C. albicans* EPM [45]. This matrix is composed by 55% protein, 25% carbohydrate, 15% lipid and 5% nucleic acids [46]. The most abundant polysaccharides included *α*-1,2 branched *α*-1,6-mannans (87%) associated with unbranched β-1,6-glucans (13%) in an apparent mannan-glucan complex, while β-1,3 glucan comprised only a small portion of the total EPM carbohydrates [46]. The presence of β-1,3 glucan has been recently reported in *C. glabrata* biofilms [47] being a key carbohydrate component in fungal cell walls [47,48]. 

### 1.4. Stages in the Development of Biofifilms

The development of a biofilm involves five stages that were clearly explained and graphed by Stoodley et al. [49], Ramage et al. [50], Mayer et al. [51] and other authors [43,52,53,54]. In stage 1, planktonic cells adhere to a biotic or abiotic surface, followed by a yeast-to-hyphal transition (Figure 1a,b). In the second stage, EPM is produced, resulting in a firmly adhered irreversible attachment. In stage 3 an early biofilm architecture is developed, while in stage 4 the biofilm reaches maturation in a three-dimensional structure (Figure 1c). Finally, in stage 5, single planktonic cells are dispersed from the mature biofilm. This dispersion is an important step in the fungal biofilm development cycle, which can induce either bloodstream or invasive fungal infections [55] leading to an increased pathogenicity [56] and high risk of mortality [57,58]. This consortium of cells offers the optimum conditions for the fungi to obtain nutrients, dispose waste products and protect from the environment [58]. Once the biofilm is formed, the sessile cells communicate with each other through quorum sensing (QS) molecules that induce the fungal population to cooperate in diverse defense behaviors such as virulence and biofilm formation [59,60,61]. The sesquiterpene farnesol and the aromatic alcohol tyrosol are the two reported natural occurring QS molecules [62,63]. The first inhibits filamentation and biofilm formation in *C. albicans* [64] while tyrosol stimulates the germ tube formation and, thus, filamentation [63].

### 1.5. Factors and Mechanisms Associated with Biofilm Resistance

One of the major characteristics of biofilms is the enhanced resistance to antimicrobial agents [65,66,67,68,69]. It has been reported that they possess about 5- to 4000-times less susceptibility to antifungals than equivalent populations of planktonic cells [60,69,70,71,72], which is a decisive factor driving therapeutic failures [73].

Several factors appear to be responsible for the resistance of biofilms [74,75,76]: (i) EPM acts as a physical barrier, preventing the entry of antifungals to biofilms, by either slowing drug diffusion or through specific sequestration mechanisms [74], with EPM components playing key roles [75]. For instance, soluble 1,3-*β* glucan, released from the fungal cell wall of *C. albicans* and *A. fumigatus* is able to sequester antifungal molecules, especially azole and polyene drugs, thus preventing their access to biofilm cells [68,76,77]; (ii) ergosterol levels are significantly lower at intermediate and mature phases of biofilms, compared to those in early phase biofilms and, therefore, those antifungals which target ergosterol have a poorer effect [78]. (iii) Although most sessile cells can show susceptibility to antifungal agents, a small sub-population of cells (persisters) stay alive [79], protected by the EPM and with a smaller oxidative imbalance compared to planktonic cells [80]. These persister cells can survive both the antifungal treatment and the immune system [81]. When the concentration of antifungal decreases, persister cells revive and repopulate the biofilm [81,82,83]. (iv) One of the major mechanisms of resistance to several classes of antifungals is the overexpression of efflux pumps [73]. These proteins imbibed in membranes are able to export the antimicrobial agents out of the cells, leading to a reduced intracellular concentration, which hinders the desired pharmacological effect. 

The expression of different genes associated with resistance occurs during biofilm formation and maturation. The most prominent classes of transporters are those belonging to the ATP binding cassette (ABC) and major facilitator superfamily (MFS) [84]. In *Candida* spp., the ABC transporters Cdr1p and Cdr2p, and the MFS transporter Mdr1p, emerged as the main contributors in biofilm-associated resistance to a range of antifungal agents, mainly to the azoles [6,85]. The expression of the transporters Cdr1p, Cdr2p and Mdr1p showed a dependency with the developmental phases of biofilms, therefore leading to different degrees of susceptibility associated with the outward transport of drugs. The overexpression of *CDR1*, *CDR2* and *MDR1* genes occurred at the early stages of biofilm formation, being the prominent mechanism for the tolerance to fluconazole, while in mature biofilms other resistance mechanisms are also involved in the diminished susceptibility to this azole drug [65,78,86].

In turn, the increase of drug resistance to antifungals, such as fluconazole and amphotericin B during biofilm maturation, seems to be associated with a significant decrease in the total ergosterol content, with changes in the expression of some *ERG* genes involved in its biosynthesis compared to planktonic cells [87]. Mutations or overexpression in the *ERG11* gene, which encodes lanosterol 14α-demethylase [88,89], as well as in other *ERG* genes such *ERG1* (encodes squalene epoxidase), *ERG3* (encodes D5,6-desaturase), *ERG7* (encodes squalene cyclase), *ERG9* (encodes squalene synthase) or *ERG25* (encodes C-4 methyl sterol oxidase) play important roles in the resistance of fungi to different antifungal agents [72,90,91,92].

### 1.6. Methodologies to Assess Antibiofilm Activity

At present, in vitro and in vivo assays are used for quantifying biofilms [93]. Several methods for the in vitro assessing of the minimum inhibitory concentrations of biofilm sessile cells have been developed. The most commonly used assay is based on a static model using a microtiter plate [94], where the microbial biomass or metabolic activity are quantitated using dyes such as crystal violet (CV), 2,3-bis (2-methoxy-4-nitro-5-sulfophenyl)-5-[(phenylamino) carbonyl]-2H-tetrazolium hydroxide (XTT), or fluorescein diacetate or resazurin [95]. Additionally, colony-forming units (CFU) counting has been extensively used [96]. Flow cytometry, using different fluorophores, can be used for determining CFU [7]. Another stain is calcofluor white (CW), which binds to 1,3 and 1,4 carbohydrate linkages and fluoresces under long-wave UV light [97]. Other biofilm devices or microscopy techniques such as light, confocal laser scanning microscopy (CLSM) and scanning electron microscopy (SEM), among others, have been extensively used [96,98,99]. To determine the inhibition of biofilm ‘formation’, compounds are incorporated during the biofilm growth phase. Instead, to assess biofilm ‘eradication’, biofilms are grown for 24 h, after which the compounds are added and biofilms are additionally incubated 24–48 h in the presence of the compounds. The minimum biofilm inhibitory concentrations (MBIC, MBIC_80_ or MBIC_50_) are the minimum concentrations resulting in 100, 80 or 50% inhibition of biofilm formation, respectively [100]. Similarly, the minimum biofilm eradication concentration MBEC, MBEC_80_ or MBEC_50_ are the minimum concentrations resulting in 100%, 80% or 50% eradication of mature biofilms, respectively [101].

The worm *Caenorhabditis elegans* is highly used for the in vivo antibiofilm effectiveness of compounds, since hyphal formation is able to kill *C. elegans* [102]. Results are expressed as percentages of living or dead worms after incubation for a given number of days [93]. Several other models such as the venous catheter model in rats, rabbit or mice [93]; urinary catheters, subcutaneous implants, denture stomatitis, and oral and vaginal mucosae in different animals, are used. All these models were thoroughly reviewed by Nett and Andes in 2015 [103].

### 1.7. Natural Products as an Important Source of Antifungal Drugs

Most antifungal agents in clinical use are natural or natural-derived compounds, mainly isolated from microbial strains [104]. For example, the polyenes nystatin and amphotericin B were isolated from *Streptomyces* spp. [105]; the spiro-diketone griseofulvin, was obtained from *Penicillum griseofulvum* [106]; the echinocandins caspofungin and anidulafungin are semisynthetic derivatives from the natural pneumocandin B, which was isolated from the fungus *Glarea lozoyensis* [107]. In turn, micafungin derived from the lipopeptide FR901379 was isolated from the fungus *Coleophoma empetri* [107], a plant pathogen associated with postharvest fruit rot in cranberries [108]. Under this scenario, it is clear that nature has provided a robust platform for finding novel scaffolds for the discovery of antifungal drugs.

Natural antifungal products were typically discovered using the traditional antifungal assays, which measure the inhibition of growth of yeasts and filamentous fungi in their planktonic state, thus free-floating in the culture medium [109]. Recent works have reported extracts either from plants [110,111,112], algae [113,114], endophytic fungi [115] or marine fungi [116], as well as secondary metabolites such as phenols [111,117], flavonoids [118], naphtoquinones [119] and terpenoids [120], which showed growth inhibitory properties of fungi in their planktonic state. Both recent and previous reviews reported several pure natural products that have shown antifungal activity assessed with this type of techniques. However, none of these compounds have become leads for the development of new antifungal drugs [121].

The aim of this present review is to provide an update (2017–May 2021) on the natural products (extracts and secondary metabolites) that possess the ability to in vitro or in vivo modulate fungal biofilms not only constituted by *C. albicans* and some non-*albicans Candida* yeasts, but also by fungi from other genera. When available, the mechanism of action of these natural products has been included. To this purpose, most data published in the literature from 2017 were collected with the objective of drawing conclusions that can be useful for future research. Previously, some reviews on this subject were published [122,123,124,125,126] by Nazzaro et al. [123] and Singla and Dubey [124], with very few references each regarding the effect against fungal biofilms after 2017.

## 2. Reported Antibiofilm Activities of EOs, Propolis and Extracts from Plants, Algae and Cyanobacteria

Different studies involving EOs with antibiofilm activity are recorded in Table 1. Peixoto et al. [127] reported that *Laurus nobilis* L. (Lauraceae) EO at 2× minimum inhibitory concentration (MIC, 1000 µg/mL) inhibited the initial adhesion of *C. albicans*, while at 2× and 4× MIC, it inhibited biofilm formation and also reduced the eradication of mature biofilms with no significant difference when compared to the positive control, nystatin. In another study, Manoharan et al. [128] screened 83 EOs against *C. albicans* biofilm formation. Six of them obtained from *Croton eluteria* (L.) W.Wright (Euphorbiaceae) (cascarilla bark), *Helichrysum coriaceum* (DC.) Harv. (Asteraceae) (helichrysum oil), *Eucalyptus globulus* Labill. (Myrtaceae), *Cymbopogon citratus* (DC.) Stapf (Poaceae) (lemongrass), *Citrus aurantiifolia* (Christm.) Swingle (Rutaceae) (lime oil) and *Coriandrum sativum* L. (Apiaceae) (coriander) were demonstrated to inhibit more than 90% of biofilm formation when tested at 0.01%. Among them, cascarilla bark and helichrysum oil and their main components, α-longipinene and linalool, significantly reduced the yeast-to-hyphal transition, adherence and biofilm formation and greatly inhibited invasive hyphal growth in the nematode *C. elegans*. Serra et al. [129] tested different commercial EOs against two *C. albicans* strains. The results showed that only *Pelargonium graveolens* L’Hér. (Geraniaceae) (geranium) and *Melisa officinalis* L. (Lamiaceae) (melissa) EOs, eradicated mature biofilms with MBEC_80_ of 22.3 and 17.9 µg/mL, respectively for geranium and of 13.3 µg/mL on both strains, for melissa.

As reported by Banu et al. [130], EOs from *Cinnamomum tamala* (Buch.-Ham.) T. Nees and Eberm. (Lauraceae) (Indian cassia), *Pogostemon heyneanus* Benth (Lamiaceae) (Indian patchouli) and *Cinnamomum camphora* (L.) J.Presl (Lauraceae) (camphor) inhibited about 54%–65% of biofilms formed by *C. albicans*, *C. glabrata* and *C. tropicalis*. In addition, the three EOs reduced the *Candida* spp. preformed biofilms, with an inhibition range of 55%–67% at their MBICs (0.5%–5% *v*/*v*); with *C. tamala* being the most active plant sp., *P. heyneanus* EO showed the maximum inhibition of yeast-to-hyphal transition. On the other hand, the EOs from *P.* *heyneanus* and *C. tamala* disrupted *Candida* spp. mature biofilms and reduced the thick aggregation of the yeast cells. This result was confirmed by the observation of a decrease of sugars present in the EPM layer.

The capacity of *Foeniculum vulgare* Mill. (Apiaceae) EO (fennel oil) to eradicate 10 strains of *C. albicans* biofilms was studied by Bassyouni et al. [131]. The MBEC_50_ of fennel oil for eradicating the 18-h-old biofilm was 2- to 16-fold of MIC, in 7/10 tested strains. Sahal et al. [132] investigated the antifungal and biofilm inhibitory effects of EOs by using *C. tropicalis* biofilms coated on different biomaterials. Treatments with 2%–8% of *C. citratus* EO coated on silicone rubber resulted in a 45%–76% reduction in biofilm formation of all the strains. Likewise, *Cuminum cyminum* L. (Apiaceae), *Citrus limon* (L.) Osbeck (Rutaceae) and *Cinnamomum verum* J.Presl (Lauraceae) EOs, were also effective in inhibiting the *C. tropicalis* biofilms in polystyrene plates at sub-MIC values. Therefore, the mentioned extracts, in particular *C. citratus* EO, could be used as an antibiofilm agent on silicone rubber prostheses and medical devices. *C. tropicalis* biofilms pose a serious risk for skin infections and may cause a shorter lifespan of the prosthesis. In an additional study, Choonharuangdej et al. [133] tested the efficacy of *C. verum* and *C. citratus* EOs for eradicating *C. albicans* biofilms established on heat-polymerized polymethyl methacrylate (PMMA) material and determined whether they were able to retard the formation of fungal biofilms and/or eradicate them. Results showed that cinnamom oil at 0.8 µL/mL (8× MIC) and lemongrass oil at 6.4 µL/mL (16× MIC), both coated on PMMA, inhibited the formation of *C. albicans* biofilms by 70.0% after 24 h of treatment. In contrast, at 8× MIC (0.8 and 3.2 µL/mL, respectively), both EOs eradicated 99% of the pre-established *C. albicans* biofilm in 1 h.

Table 2 shows the relevant studies on antifungal propolis with antibiofilm activity. Galletti et al. [134] evaluated the activity of green propolis collected in Paraná (Brazil) against *Fusarium* spp. biofilms that frequently cause disseminated infections in immunocompromised patients, with a high rate of mortality [135]. In the mentioned work, the authors used clinical isolates of *F. oxysporum*, *F. solani* and *F. subglutinans* and a standardized *F. solani* strain. The used propolis proved to be efficient to reduce both the total biomass (assessed with CV dye) and the number of viable cells (quantified with XTT) for all evaluated isolates. In addition, the CW fluorescence assay showed that biofilm structure was lost, leaving only isolated damaged cells. In a recent paper, Martorano Fernandes et al. [136] evaluated the inhibitory effects of Brazilian red propolis on *C. albicans* and a co-culture of *C. albicans*-*C. glabrata* biofilms. Metabolic activity determined by 3-(4,5-dimethylthiazol-2-yl)-2,5-diphenyltetrazolium bromide (MTT) assay, and cell viability assessed with CFU counts and surface roughness (optical profilometry) were evaluated. Results showed that red propolis had high inhibitory mono sp-biofilm effects but low activity against co-cultured two spp. biofilm formation, compared to chlorhexidine. The surface roughness (Sa parameter) within the mono-sp. and the co-culture biofilms statistically differed among groups.

Table 3 shows the different studies on extracts from plants, lichens, algae and cyanobacteria with antibiofilm activity.

Hydroethanol extracts of leaves, pulps, seeds and barks of several *Eugenia* spp. (Myrtaceae) such as *E. leitonii* Legrand, *E. brasiliensis* Lam., *E. myrcianthes* Nied. and *E. involucrata* DC. were tested for their capacity to eradicate mature *C. albicans* biofilms [137]. Among them, extracts from *E. leitonii* seeds and *E. brasiliensis* seeds and leaves reduced *C. albicans* biofilm viability by 54%, 54% and 55% at 156.2, 156.2 and 312.5 µg/mL (10× MIC), respectively, with better activity than that of nystatin which showed a 42% reduction. At these concentrations, all extracts caused damage to biofilm architecture and integrity, which could be observed by SEM. Alizadeh et al. [138] determined by CV assay that the ethanol extract of *Malva sylvestris* L. (Malvaceae) root at 0.78 and 1.56 mg/mL (MIC and 2× MIC, respectively) reduced *C. albicans* biofilm growth. By light microscopy, the authors observed that the extract was able to decrease biofilm thickness and cellular density. Silva et al. [139] determined that the hydroethanol extract of *Anadenanthera colubrina* Vell. Brenan (Fabaceae) barks, from the Caatinga biome (Brazil), showed the capacity to eradicate mature biofilms formed by four *C. albicans* strains, and one of each *C. parapsilopsis* and *C. krusei*, causing, in the last two, a 100% decrease of biofilms at 500× MIC. The Algerian *Clematis flammula* L. (Ranunculaceae) ethanol leaves (CFL) and *Fraxinus angustifolia* Vahl. (Oleaceae) leaves (FAL) and bark (FAB) extracts at 500 μg/mL showed 36.8%, 62.4% and 54.8% inhibition of *C. albicans* biofilm formation, respectively, which was probably related to the disruption of the cell surface hydrophobicity (CSH) and to the inhibition of germ tube and hyphae formation [140]. After four h incubation, 66.32% of hyphal form was seen in the control group, while 3.96%, 2.11% and 1.65% of hyphae were formed in the presence of CFL, FAL and FAB, respectively [140]. As reported by Dwivedi et al. [141], the *Hibiscus sabdariffa* L. (Malvaceae) flower DMSO extract inhibited the yeast-to-hypha transition with hyphae showing morphological changes, and also adherence of *C. albicans* cells (80% at 6.25 mg/mL). It also inhibited biofilm formation as well as disrupted the pre-formed *C. albicans* biofilm by 50% when tested at 3.12 mg/mL. The hexane extract of purple leaves from Orthosiphon aristatus (*Blume*) Miq. (Lamiaceae) was tested on different biofilm stages. [142]. Treatments of *C. albicans* with the extract at 2 mg/mL, showed a 69.2% decrease in cell viability (assessed with MTT) at the adhesion stage, while fluconazole at 6 µg/mL caused a 54.7% reduction. It is important to highlight that a 50% reduction in cell density compared to the negative control was observed in the presence of 1.3 mg/mL of the extract, as determined by the CV assay. At the development stage, a 57.1% and 57.3% inhibition on *C. albicans* growth, in the presence of the extract and fluconazole respectively, was observed. A low inhibition of approximately 20% was observed on mature biofilms after treatment with both the extract and fluconazole [142]. The ability to inhibit the formation (named in this paper ‘anti-maturation’) or to eradicate preformed 24 h-old *C. albicans* biofilms (named ‘antibiofilm’) were evaluated for 38 lichen acetone extracts, by XTT assay [143]. Among them, eleven extracts showed antibiofilm activity, with seven displaying both anti-maturation and antibiofilm properties. Of them, extracts from *Evernia prunastri* (L.) Ach (Parmeliaceae) and *Ramalina fastigiata* (Pers.) Ach. (Ramalinaceae) were the most promising ones, with half inhibitory concentration (IC_50_) values <4 µg/mL for anti-maturation. *E. prunastri*, *Cladonia uncialis* (L.) Weber ex F.H.Wigg (Cladoniaceae), *R. fastigiata* and *Xanthoparmelia conspersa* (Ehrh. ex Ach.) (Parmeliaceae) extracts showed IC_50_ values <10 µg/mL for antibiofilm eradication. Cepas et al. [144] tested 675 hexane, ethyl acetate and methanol extracts of 225 microalgae and cyanobacteria against *C. albicans* and *C. parapsilopsis* biofilms, which were inhibited by 308 extracts. Among the 11 phylum, the lowest activity was reported for Euglenophyta, with MBIC_50_ and MBIC_90_ of 8 and 16 µg/mL, respectively; Cryptophyta showed MBIC_50_ and MBIC_90_ values of 8 and 128 µg/mL while Glaucophyta presented MBIC_50_ and MBIC_90_ values of 8 and 256 µg/mL, respectively, against *C. albicans*. Instead, Rhodophyta spp. showed MBIC_50_ and MBIC_90_ values of 64 and 512 µg/mL, respectively, against *C. parapsilopsis*.

## 3. Reported Antibiofilm Activities of Pure Natural Compounds

Pure natural compounds with antibiofilm activity are summarized in Table 4 and detailed below. Liu et al. [145] demonstrated that the formyl-phloroglucinol meroditerpenoid, eucarobustol E (EE), isolated from *Eucalyptus robusta* Sm. (Myrtaceae), suppressed 73% of *C. albicans* biofilm formation at 32 µg/mL, destroyed nearly all mature biofilms (92%) at 128 µg/mL, blocked the yeast-to-hyphal transition and reduced the CSH at 16 µg/mL. EE downregulated the expression of genes involved in hyphal growth (EFG1, CPH1, TEC1, EED1, UME6, and HGC1), cell surface proteins (ALS3, HWP1, and SAP5) and in ergosterol biosynthesis (ERG6, ERG13, ERG252, ERG11, ERG10, and ERG7). This activity resulted in the reduction of ergosterol, which alters cell membrane functions, leading to cell death. In turn, EE upregulated the farnesol-encoding gene DPP3, which negatively regulated biofilm formation. According to the authors, EE differs from clinical antifungal agents in their antibiofilm mechanisms and so it is certainly worth considering for further development as an antifungal drug. Shi et al. [146] reported that berberine (BBR) inhibited biofilm formation of 13 strains of *C. tropicalis* and one strain of each *C. albicans*, *C. parapsilosis* and *C. glabrata* with MBICs ranging from 64 to 256 µg/mL. The mRNA expression of ERG11 and of the efflux proteins CDR1 and MDR1 were 1.43–2.10-fold upregulated by BBR at 16 µg/mL. Behbehani et al. [147] found that the lignan magnolol (2-(2-hydroxy-5-prop-2-enylphenyl)-4-prop-2-enylphenol), isolated from *Magnolia officinalis* Rehder and E.H. Wilson (Magnoliaceae), showed strong antibiofilm formation activity against *C. albicans*, *C. dubliniensis* and *C. glabrata*, with 69.5%, 46.7% and 35.6% at 32 µg/mL, respectively, as determined by MTT assay. Six EO’s components such as thymol, carvacrol, cinnamaldehyde, citral, menthol and eugenol were tested by Kumari et al. [148]. These compounds proved to be effective on biofilm formation and on preformed *C. neoformans* and *C. laurentii* biofilms in the following order: thymol > carvacrol > citral > eugenol = cinnamaldehyde > menthol with MBIC_80_ ranging from 32 to 128 µg/mL and MBEC_80_ ranging from 64 to 256 µg/mL, determined by XTT assay. SEM and CLSM showed the absence of EPM, reduction in cellular density and alteration of the surface morphology of biofilm cells. Cryptococcosis is a systemic infection [149] very difficult to treat due to the ability of these fungi to form biofilms resistant to standard antifungal treatments. Kumari et al. [150] deepened the study of the *C. neofomans* antibiofilm activity of thymol (16 µg/mL), carvacrol (32 µg/mL) and citral (64 µg/mL) using field emission scanning electron microscopy (FE-SEM), atomic force microscopy (ATM) and Fourier transform infrared spectroscopy. The three terpenes appear to act through the interaction with ergosterol or the inhibition of its biosynthesis, and the disruption of the biofilm cell surface, with pore formation and efflux of the K+/intracellular content. Morphological changes and qualitative/quantitative alterations in the EPM and in cellular components of *C. neoformans* biofilm cells were also observed. The terpenes-treated cells showed 35%−45% reduction in total carbohydrates, with variation in the type of glycosyl residues. Li et al., showed that the eudesmane sesquiterpene ent-isoalantolactone (ent-iLL) showed inhibition of the yeast-to-hyphal conversion of a mutant of *C. albicans* in assays performed in liquid and solid media at 8 µg/mL and 4 µg/mL, respectively [151]. In addition, ent-iLL at 16 µg/mL reduced the presence of ergosterol in the membrane, through inhibiting the activity of Erg11 and Erg6 [151]. The polyphenol curcumin (Cur) was evaluated for their antibiofilm properties against *C. albicans* by Alalwan et al. [152]. The MBIC_80,_ was 200 µg/mL, as determined by XTT assay. Furthermore, Cur at 50 µg/mL was able to decrease *C. albicans* adhesion to a PMMA denture base material, an effect that could be enhanced by pre-treatment of the yeasts with the polyphenol. Regarding its molecular effects, Cur down-regulated the adhesin ALS3, with minimal effect on its related ALS1. On the contrary, the clustered aggregative and flocculation genes AAF1, EAP1, and ALS5 transcripts were up-regulated. Three gingerols (6-, 8- and 10-gingerols) and three shogaols (6-, 8-, and 10-shogaols) isolated from *Zingiber officinale* Roscoe (Zingiberaceae) showed antibiofilm and anti-virulence activities against a fluconazole-resistant *C. albicans* strain. Results showed that only 6-shogaol at 10 µg/mL and 6- and 8-gingerols at 50 µg/mL, significantly reduced the *C. albicans* biofilm formation, suggesting that the increase in the length of the side chain decreased the activity. CLSM showed that biofilms treated with 6-gingerol and 6-shogaol were reduced in density and in thickness. In addition, both compounds inhibited hyphal growth in embedded colonies and free-living planktonic cells, and prevented cell aggregation, which was confirmed by SEM. Both entities significantly altered the expressions of some hypha-specific (HWP1 and ECE1), biofilm-related (HWP1 and RTA3) and multidrug transporter (CDR1 and CDR2) related genes [153]. Yan et al. [154] reported that the 1,4-naphtoquinone derivative shikonin (SK), at 32 µg/mL, inhibited almost totally C. albicans biofilm formation and eradicated the preformed mature biofilms, as evidenced with XTT assay and confirmed by CLSM. SK inhibited hyphae formation, showing a complete inhibition at 0.5 µg/mL in Lee´s medium and reduced CSH by 70.3% at 2 µg/mL. Several hypha and adhesion specific genes such as of ECE1, HWP1, EFG1, CPH1, RAS1, ALS1, ALS3 and CSH1 were downregulated while TUP1, NRG1 and BCR1 were upregulated by SK. In addition, SK induced the production of farnesol at 8 µg/mL, which enhanced its antibiofilm activity. Saibabu et al. [155] showed that different *C. albicans* strains treated with 62.5 μg/mL of vanillin showed few or no adherence to buccal epithelial cells. By MTT assay, it was observed the adherence of *Candida* cells to polystyrene surface was reduced by 52%, the biofilm formation was decreased by 49%, and the mature biofilm eradication decreased by 52%. At 125 µg/mL, vanillin protected *C. elegans* against *C. albicans* infection and enhanced its survival [155]. Kischkel et al. [156] evaluated farnesol on preformed *F. solani* complex biofilms particularly formed by Fusarium keratoplasticum, which is the most prevalent fungi related to biofilm formation in hospital water systems and in internal pipelines. Farnesol showed activity against *F. keratoplasticum* preformed biofilms, and was effective also during its formation at different times (at adhesion and at 24, 48 and 72 h) with a complete inhibition at 700 µM, as assessed by counting the CFU number and by CV and XTT assays.

A recent report from Wang et al. [157] described the isolation of 14 new terpenoids from the liverwort *Heteroscyphus coalitus* (Hook.) Schiffner (Geocalycaceae), including eight ent-clerodane diterpenoids, four labdane diterpenoids, heteroscyphsic acids A-I, heteroscyphins A-E, a harziane type diterpenoid and one guaiane sesquiterpene together with a known ent-junceic acid. At 4–32 μg/mL most of these compounds inhibit hyphal and biofilm formation of an efflux pump deficient strain of *C. albicans*, but not of the wild type of strain. The most effective molecule was heteroscyphin D, which suppressed the ability of *C. albicans* to adhere to A549 cells and to form biofilms with a complete inhibiton at 8 μg/mL, determined by XTT assay. In addition, this compound was able to modulate the transcription of related genes in this fungus, as described in Table 4. Das et al. [158] showed that artemisinin (Ar), the sesquiterpene lactone isolated from *Artemisia annua* L. (Asteraceae), and scopoletin (Sc), the 7-hydroxy-6-methoxy coumarin present in several spp., were tested on mature biofilms of different *albicans* and non-*albicans Candida* strains. The results demonstrated that Ar was more active than Sc in disrupting the preformed EPM-covered biofilm structure and in killing the sessile cell population at their respective MBEC_10_. In the same year, Lemos et al. [159] demonstrated that Sc, at its MIC (50 µg/mL), was able to reduce the preformed biofilms of a resistant *C. tropicalis* strain in 68.2%, and to inhibit the biofilm formation. Kipanga et al. [160] showed that the drimane sesquiterpene dialdehydes warburganal and polygodial, obtained from *Warburgia ugandensis* Sprague (Canellaceae), inhibited *C. albicans* biofilm formation with MBIC_50_ of 4.5 and 10.8 µg/mL, respectively, and with MBIC_50_ of 49.1 and 50.6 µg/mL, respectively, against *C. glabrata*. Regarding biofilm eradication, warburganal and polygodial showed MBEC_50_ of 16.4 and 16.0 µg/ml, respectively, against *C. albicans* but did not inhibit *C. glabrata* biofilm eradication. The higher potency of warburganal over polygodial for inhibiting biofilm formation and eradication could be attributed to the hydroxyl group present at position C-9, that differentiates both sesquiterpenes. The triterpenoid saponins gypenosides, isolated from *Gynostemma pentaphyllum* (Thunb.) Makino (Cucurbitaceae), showed MBIC_80_ > 128 µg/mL against two fluconazole-resistant strains of *C. albicans*, as determined by XTT. No significant reduction in the density and in the length of the hyphae were observed [161]. Zhao et al. [162] showed that turbinmicin, a highly functionalized polycyclic compound isolated from the marine microbiome, completely disrupted extracellular vesicle (EV) delivery, during biofilm growth at 16 µg/mL, and this impaired the subsequent assembly of the biofilm EPM. *C. albicans* biofilm EVs have a pivotal role in EPM production and biofilm drug resistance [163]. This property was observed by a combination of flow cytometry, image confirmation, and fluorescence sensitivity on *C. albicans*, *C. tropicalis*, *C. glabrata*, *C. auris* and *A. fumigatus*. By SEM, it was determined that turbinmicin at 2.5 µg/mL eliminated EPM, thus rendering the drug-resistant biofilm communities susceptible to the antifungal effects of TBM itself, as well as to clinical antifungal agents. Zainal et al. [164] demonstrated that allicin, the organosulfur compound obtained from *Allium sativum* L. (Amaryllidaceae), was able to eradicate 50% of *C. albicans* biofilm formed on self-polymerized acrylic resin when administered at a sub-MIC concentration of 4 µg/mL. Feldman et al. [165] reported that cannabidiol (CBD) obtained from *Cannabis sativa* L. (Cannabaceae) exerted an inhibitory effect on biofilm formation with a MBIC_90_ of 100 µg/mL. At 25 µg/mL, the metabolically active cells (assessed by MTT) in 24, 48 and 72 h-biofilms decreased by 48%, 64% and 87%, respectively. At 1.56 and 3.12 µg/mL, mature biofilms decreased by 28% and 44%, respectively. Furthermore, CBD reduced the thickness of fungal biofilm and EPM production accompanied by a downregulation of genes involved in EPM synthesis. At 25 µg/mL, CBD inhibited 90% of the ATP synthesis and enhanced mitochondrial membrane potential and ROS levels. At ¼ MBIC_90_, CBD produced upregulation of yeast-associated genes and downregulation of hyphae-specific genes. As reported by Ivanov et al. [166], camphor inhibited more than 50% of the biofilm biomass in *C. albicans* strains at 62–250 µg/mL, while in *C. tropicalis,* the inhibition was achieved at 175 µg/mL. On the other hand, eucalyptol showed the same effect in the tested *C. albicans* strains at higher concentrations (>3000 µg/mL) and in *C. tropicalis*, *C. parapsilosis* and *C. krusei* at >1000 µg/mL [166]. Camphor, applied at 125 µg/mL, reduced ROS generation in one strain of *C. albicans*, although eucalyptol failed to exert this effect. The compounds did not interfere with ERG11 expression. *Neosartorya fifischeri* antifungal protein 2 (NFAP2), a novel member of small cysteine-rich and cationic antifungal proteins from filamentous ascomycetes (crAFPs), showed low activity against *C. auris* biofilms [167]. When NFAP2 was tested in combination with clinical antifungals, an enhancement of the activity was observed. The results of combinations were not included in this review.

Although the main objetive of this review is to provide published data on the in vitro or in vivo antibiofilm activity of natural products, mention should be made to the method reviewed by Jha et al., 2020 [17]. These authors performed a multiple target-based structural bioinformatic study to recognize molecules that have the capacity of targeting proteins with a vital role in wall synthesis (FKS2 and FKS1), ergosterol synthesis (ERG11, ERG1, ERG24 and ERG3), and drug transport, such as Flu1 and the kinases CST20, HST7 and CEK1 involved in CHP1 pathway. The Cph1 transcription factor is involved in biofilm and pseudohyphae formation [168]. According to the results obtained, 2-*O*-prenylcoumaric, acid, 4-coumaryl acetate, coniferaldehyde, coniferyl alcohol, cycloserinehybrid and 2-coumaric acid targeted all proteins, while 110 molecules bound to at least four of them [17]. Among the evaluated compounds, vanillin showed interactions with ERG11, ERG1, ERG24, FKS2, CST20, HST7 and CEK1 while BBR targeted all of these proteins except Flu1 [17]. Thymol interacted to all proteins, except ERG24 and ERG1, and carvacrol bound to ERG24, ERG1 HST7, CST20, CEK1 and Flu1. Likewise, eugenol targeted ERG24, ERG1, FKS1, FKS2, FUR1 and Flu1 and cinnamaldehyde interacted with all of the proteins except FKS1, FKS2, FUR1 and Flu1. These data would explain, at least in part, the antibiofilm activity of the mentioned small molecules, which has been reported above and also in Table 4, while opening the possibility to test experimentally the effects on biofilms of the rest of the ligands.

## 4. Reported Antibiofilm Activities of Nanosystems Containing Natural Products

The published literature related to the antibiofilm properties of nanosystems are shown in Table 5. Quatrin et al. [169] prepared nanoemulsions (NE) containing 5% *E. globulus* EO and 2% sorbitan monooleate (Span 80) with the aqueous phase containing 2% Tween 80. EO-NE were tested for their capacity to inhibit biofilm formation of three *Candida* spp. in high density polyethylene substrates. EO-NE at 22.5 mg/mL reduced *C. albicans*, *C. tropicalis* and *C. glabrata* biofilm formation by 84.5%, 61.3% and 84%, respectively. The biofilms were quantified by CV staining and corroborated by ATM and the fluorescence CW technique.

Due to *M. alternifolia* EO (tea tree oil (TTO)) showed antibiofilm activity against *C. albicans* spp. in previous studies but possesses poor solubility and high volatility [170], Souza et al. [171] prepared TTO-nanoparticles and tested them against *C. albicans*, *C. glabrata*, *C. parapsilopsis*, *C. membranafaciens* and *C. tropicalis* biofilms. The results obtained showed that TTO and TTO-nanoparticles decreased by 67% and 72% respectively the *C. albicans* and *C. glabrata* biofilm biomass visualized with CV and confirmed by CW and ATM. The antibiofilm activity was more evident against *C. glabrata*. TTO-NP decreased EPM and proteins in biofilms and, therefore, TTO-NP can penetrate more easily through the EPM, releasing the TTO into the biofilm and resulting in a better antimicrobial activity. TTO-NPs can disturb the fungal membrane by blocking the respiratory chain through the inhibition of the enzyme succinate dehydrogenase of the internal fungal cell mitochondrial membrane.

Considering that free Cur at 200 μg/mL inhibited almost completely the *C. albicans* biofilm formation, Ma et al. [172] prepared Cur-chitosan nanoparticles (CSNP). However, CSNP-Cur exhibited a slightly less inhibitory effect than free Cur. On the contrary, 400 μg/mL of CSNP-Cur eradicated the preformed *C. albicans* biofilms by 91.38%, being more effective than free Cur. SEM and CLSM showed that CSNP-Cur reduced polymicrobial biofilm thickness as well as killed microbial cells embedded in biofilms on silicone surfaces. In a recent paper, Gumus et al. [173] prepared nanoparticles of juglone (JU) encapsulated in poly d,l-lactic-co-glycolic acid (PLGA). As determined by plate count technique, these PLGA-JU nanoparticles completely inhibited biofilm formation and pre-established biofilms, at doses equivalent to 1.25 and 0.625 mg/mL of JU, respectively, whereas fluconazole did not cause a significant inhibition, even at the highest dose applied (10 mg/mL). PLGA-JU were more active than free JU and fluconazole. The inhibition of biofilm formation seems to be due to PLGA-JU reducing the cell adhesion, and the number of viable cells and altered membrane structures at both mentioned doses.

Rajasekar et al. [174] synthesized a nanocomplex formed by the surfactant sophorolipid (SL) derived from non pathogenic yeasts such as *Starmerella*/*Candida bombicola* and Cur, and tested it against *C. albicans* biofilms. Sub-inhibitory concentrations of Cur-SL (9.37 μg/mL) significantly decreased fungal adhesion to glass coverslips, as well as biofilm development, maturation, and filamentation. Concomitantly, a significant downregulation of a select group of biofilm adhesins and hyphal regulatory genes was observed.

## 5. Discussion

In this review, the natural products that demonstrated activity against fungal biofilms from 2017 to May 2021 were collected, with the aim of offering an overview of the progress made in this area to curb difficult-to-eradicate fungal infections. The type of natural products that showed better antibiofilm activities, the fungal species of the target biofilms, the type of assays used and the mechanisms of action were analyzed in order to detect the advances performed in this period that can be the basis of future works.

Regarding the type of natural products that showed antibiofilm activities, 17 of the 42 manuscripts involved natural mixtures such as EOs (seven manuscripts) propolis (two manuscripts) or extracts prepared with solvents (total eight manuscripts), six of them from plants, one from lichens and one from microalgae or cyanobacteria. Two papers dealt with nanosystems including EOs. The antibiofilm activities of the seven EOs do not add a great novelty to the already known antibiofilm activity of these products. However, the nanosystems prepared with EOs open a great avenue for further research. In addition, the paper authored by Quatrin et al. [169], which tested a nanosystem with *E. gobulus* EO (EO-NE), not only used *C. albicans* as in most publications on antibiofilm EOs, but also used *C. tropicalis*, *C. glabrata* and *C. auris* although the EO-NE was devoid of activity in the latter sp. [169]. Considering that *C. glabrata* is a fungus intrinsically resistant to most antifungal drugs [175], the activity found against this fungus is encouraging for further research. With respect to extracts obtained with solvents from different spp., no preference for a botanical family was observed, and in most cases the antibiofilm activities should be deepened since the mechanism to achieve this effect was not analyzed.

Twenty three of the 42 retrieved articles tested pure natural compounds, twenty of them alone and three included in nanosystems. Regarding the main type of pure compounds that showed a capacity for inhibiting biofilms on its own, nine of them were terpenes with four monoterpenes derivatives, four sesquiterpenes and one diterpene, which is in accordance with previous reports [124]. Two manuscripts studied the coumarin Sc, and one evaluated dammarane-type glycosides (gypenosides). A phloroglucinol, an alkaloid, a neolignan and some diphenols were investigated along with four compounds belonging to other types of chemical families. Cur was included in two nanosystems. Most of the compounds (21 compounds) were derived from plants, with one compound obtained from fungi (farnesol) and one obtained from a sea squirt microbiome (turbinmicin).

With respect to the fungal spp. tested in the 42 papers included in this review, 38 of these involved *Candida* spp. biofilms, two comprised *Fusarium* spp., two involved *Cryptococcus* spp. and one, *A. fumigatus* plus *Candida* spp. Among *Candida* biofilms, 23 studies included only *C. albicans* and 12 used different non-*albicans Candida* spp., but also involved *C. albicans*. Two papers used only *C. tropicalis* biofilms. Unfortunately, only two papers used *C. auris* among the non-*albicans Candida* spp. The newly described yeast *C. auris* has emerged as a resistant fungal pathogen responsible for hospital outbreaks, especially in intensive care units [176], leading to high mortality rates [177]. *C. auris* is at present a worrisome healthcare problem and new antifungals that overcome its resistance are highly needed [177]. The protein NFAP2 and the highly functionalized polycyclic turbinmicin completely disrupted EV delivery during *C. auris* biofilm growth [164]. Turbinmicin also showed activity against *A. fumigatus* biofilms.

Regarding anti-*C. neoformans* and *C. laurentii* biofilms, only thymol, carvacrol and citral proved to be effective for inhibiting the formation and eradication of the mentioned biofilms; however, importantly, their mechanisms of action were studied [150,152]. These are significant findings, since this fungus can colonize the central nervous system, highlighting its significance as a critical pathogenic yeast.

Propolis from Brazil and farnesol were the sole natural samples which showed a capacity for inhibiting *Fusarium* spp. This mold causes mildly superficial to fatally disseminated fungal infections, being the second most common mold causing opportunistic invasive infections after *Aspergillus* [178]. Keratitis is still the most common infection produced by *Fusarium* spp. [75,179]. The 2005–2006 outbreak of keratitis due to contact lens infections was attributed partly to the ability of *F. keratitis* to form biofilms [180,181].

Regarding the type of tests performed, most papers used only in vitro assays. Of them, twelve articles studied the inhibition of cell-to-hyphal transition or cell adhesion, 31 reported inhibition of biofilm formation, and 25 described eradication capacity.

It is worth taking into account that for the antibiofilm compounds to be useful in clinics, it is crucial to determine whether a drug is able to penetrate and eradicate the pre-formed biofilms [102]. The fact that the reported works have not assessed the biofilm eradication capacity but only inhibitory effects on biofilm formation might contribute to a poor correlation between biofilm susceptibility and clinical outcomes.

Only 15 papers deepened the study by giving evidence of the mechanisms of action. Of them, 11 were performed with pure compounds, three with nanosystems, and only one with an extract. It is important to highlight that sometimes the mechanisms of antibiofilm action were demonstrated to be multifactorial [150,182].

Only five of the 42 analyzed papers used in vivo assays, of which four were performed with *C. elegans* and only one, used rats. This is worrying since animal models of *Candida* biofilm infections are relevant for identifying novel antifungals useful in clinics [105].

## 6. Conclusions and Perspectives

From 2017 to May 2021, there has been an active research on natural products with antifungal biofilm activity. It is clear that great progress has been made and that the newly discovered natural antibiofilm agents could provide novel agents for biofilm-associated infections. Of them, the protein NFAP2 and the highly functionalized polycyclic turbinmicin have demonstrated interesting antibiofilm properties and deserve further research.

However, several types of studies must be deepened. For example, fungal biofilms different from those of *Candida* spp. should be prepared and used as targets. Among them, it is necessary to investigate the behavior of natural samples on filamentous fungal biofilms, which are scarcely studied in comparison to *Candida* spp. such as those of the genera *Aspergillus*, *Fusarium* and *Trichophyton* [183,184].

To be useful for future development, all papers should analyze biofilm eradication capacities in addition to the study of the inhibition of biofilm formation and other properties. Besides, in vivo assays should be included in all papers dealing with activity against biofilms.

It is worth taking into account that several clinical trials involving natural antibiofilm agents are in progress, and some of them exhibited promising perspectives, as recently reviewed by Lu et al. [185].

## 7. Materials and Methods

The search for suitable papers was performed in electronic databases by using the following keywords: “biofilm”, “fungal infections”, “sessile cells”, “secondary metabolites”, “natural products”, “repurposing”, “antifungal drugs”, and “antibiofilm”. Additional papers matching the search criteria were included after surveying the references from the selected articles.

The information gathered was divided into three groups: (I) natural extracts including EOs, propolis and extracts from plants, lichens, algae and cyanobacteria, (II) pure natural compounds, and (III) nanosystems. This last section was sub-divided by EOs included in nanosystems and pure natural compounds included in nanosystems.

## Figures and Tables

**Table 1:** More relevant results of essential oils (EOs) showing a capacity for inhibiting fungal biofilms.

Year	Essential Oil From	Fungal spp. Biofilm	In Vitro Type of Studies				Reference
			Inhibition of Cell Adhesion or Hyphal Formation	Inhibition of Biofilm Formation	Eradication of Mature Biofilms	Mechanism of Action or In Vivo Assays	
2017	*Laurus nobilis*	*C. albicans* ATCC 60193; CBS 562; *C. tropicalis* ATCC 750; CBS 94 and *C. krusei* ATCC 3413; CBS 73	*L. nobilis* EO at 1000 µg/mL inhibited the initial adhesion of *C. albicans* biofilms	At 1000 and 2000 µg/mL, the EO showed significant inhibition of biofilm formation	*L. nobilis* EO at 1000 and 2000 µg/mL reduced the amount of mature biofilms		[127]
2017	83 EOs	*C. albicans* ATCC 10231, 18804, 24433 and DAY185	*Croton eluteria* reduced the *Candida* adherence by 75%	Six EOs, *C. eluteria*, *Helichrysum coriaceum*, *Eucalyptus globulus*, *Cymbopogon citratus* and*Coriandrum sativum* inhibited 90% of biofilm formation		In vivo assay: *C. eluteria* EO diminished *Caenorhabditis elegans* virulence	[128]
2018	12 EOs	*C. albicans* 135, BM2/94 and NYCY 1363			Out of the 12 EOs tested, only *Pelargonium graveolens* and *Melissa officinalis* eradicated mature biofilms		[129]
2018	*Pogostemon heyneanus*, *Cinnamomum tamala***and ***Cinnamomum camphora*	*C. albicans* ATCC 90028; *C. glabrata* MTCC 6507 and *C. tropicalis* MTCC 310	The three EOs produced a reduction in the hyphal formation with *P. heyneanus* EO showing the maximum inhibition		*P. heyneanus* and *C. tamala* disrupted mature biofilms	*Candida* biofilms EPM was reduced. A large reduction of sugars was observed	[130]
2019	*Foeniculm vulgare* EO (fennel oil)	10 isolates of *C. albicans*			The MBEC_50_ of fennel oil for 7/10 tested strains was 2-to 6-fold the MIC		[131]
2020	*Cymbopogon citratus*, *Cuminum cyminum*, *Citrus limon* and *Cinnamomum verum*	*C. tropicalis* isolates T26, U7 and V89		*C. citratus* EO reduced biofilm formation of all *C. tropicalis* tested strains. *C. limon* and *C. cyminum* EOs showed minor effects			[132]
2021	*Cymbopogon citratus* and (lemongrass) *Cinnamomum verum* EOs (cinnamom)	*C. albicans* ATCC 10231 biofilms, coated on polymethyl methacrylate (PMMA) resin		*C. verum* EO at 8× MIC and *C. citratus* EO at 16× MIC, precoated on PMMA, inhibited *C. albicans* biofilm formation by 73% and 68%, respectively	At 8× MIC, both EOs eradicated totally the pre-established fungal biofilms in 1 h		[133]

ATCC: American type culture collection (Manassa, VA, USA); CBS: Netherlands Collection- Central Bureau voor Schimmelcultires; EPM: exopolysaccharide matrix; EO: essential oil; MBEC: minimum biofilm eradicating concentration; MTCC: Microbial type culture collection and Gene Bank, Chandigarh, India; NCYC: national collection of yeast cultures, Swindon, Wiltshire, UK; MIC: minimum inhibitory concentration. Other culture collection acronyms can be found in the respective reference.

**Table 2:** More relevant results of propolis extracts showing a capacity for inhibiting fungal biofilms.

Year	Extracts	Fungal Biofilms spp.	In Vitro Type of Studies			Reference
			Inhibition of Cell Adhesion or Hyphal Formation	Biofilm Formation	Eradication of Mature Biofilms	
2017	Propolis from Paraná state (Brazil) diluted in EtOH (PE) quantified in its total phenol content	*Fusarium* spp. isolated from onichomycoses and deposited in UEM; *F. oxysporum* FO42; *F. solani* FS04 and ATCC 36031 and *F. subglutinans* (FSub39)			The total mass of biofilms and the number of viable cells were significantly reduced by PE	[134]
2020	EtOH-H_2_O extract of red propolis (RPE) from Paraiba state, Brazil	*C. albicans* ATCC 90028 mono sp. biofilm and *C. albicans*-*C. glabrata* ATCC 2001 co-cultures biofilms		RPE at 3% showed a high and low inhibitory capacity of inhibiting the formation of mono sp.- and co-cultured two spp. –biofilms, respectively		[136]

ATCC: American type culture collection (Manassas, VA, USA); EtOH: ethanol; UEM: mycological collection of the laboratory of medical mycology of the Universidade Estadual de Maringá, Brazil.

**Table 3:** More relevant results of extracts from plants, algae and cyanobacteria showing a capacity for inhibiting fungal biofilms.

Year	Extracts Source and Solvent	Fungal Biofilms spp.	In Vitro Type of Studies			Reference
			Inhibition of Cell Adhesion or Hyphal Formation	Biofilm Formation	Eradication of Mature Biofilms	
**PLANT EXTRACTS**						
2017	*Eugenia leitonii*, *E. brasiliensis*, *E. myrcianthes*, *E. involucrata* leaf, pulp, seed and bark EtOH-H_2_O extracts	*C. albicans* ATCC 90028			Treatment with *E. leitonii* seed and *E. brasiliensis* seed and leaf extracts at 10 × MIC, reduced *C. albicans* biofilm viability	[137]
2017	*Malva sylvestris root EtOH extract*	*C. albicans* ATCC 10231	*M. sylvestris* extract at 0.19 mg/mL (1/4 MIC), down-regulated the expression of the hypha-specific gene *HWP1*	*M. sylvestris* EtOH extract at 0.78 and 1.56 mg/mL (MIC and 2× MIC) reduced biofilm formation		[138]
2019	*Anadenantera colubrina* bark EtOH-H_2_O extract	*C. albicans* ATCC MYA2876, ATCC 90028 and a clinical isolate; *C. parapsilopsis* ATCC 22019 and *C. krusei* ATCC 6258			*Candida* biofilms at 500× MIC underwent a decrease in the number of CFU/mL. Biofilm structural alterations and cellular destruction were observed, being *C. parapsilosis* and *C. krusei* the most affected biofilms.	[139]
2020	*Clematis flammula* fresh leaves (CFL) and *Fraxinus angustifolia* fresh leaves (FAL) and bark (FAB) EtOH extracts	*C. albicans* ATCC 10231	CFL, FAL and FAB produced a very low germ tube formation of 7.57, 2.29 and 1.17%, respectively in comparison to a growth of 50.89% for the control group	The extracts inhibited biofilm formation with MBIC_50_ = 250 μg/mL for FAL and 500 μg/mL for FAB, while CFL showed a MBIC_50_ > 1000 μg/mL		[140]
2020	*Hibiscus sabdariffa* flower (Hs) DMSO extract	*C. albicans* isolated from vulvo-vaginal candidiasis	Hs extract inhibits the yeast-to-hyphal transition and biofilm adherence (50% at 1.5 mg/mL and 80% at 6.25 mg/mL)	Fungal cells incubated with Hs extract at 2.5 mg/mL (½ MIC), inhibited the biofilm maturation and, thus, the biofilm formation	Hs extract eradicated *C. albicans* biofilms at 3.12 mg/mL. In vivo assay with *Caenorhabtidis elegans* showed that Hs decreased the CFU of *C. albicans* i	[141]
2020	Orthoshipon aristatus purple leaf *n*-hexane extract	*C. albicans* ATCC 10231	O. aristatus extract at 2 mg/mL reduced the adhesion of *C. albicans* cells	Mild inhibition of *C. albicans* growth at the biofilm development stage at 2 mg/mL		[142]
**LICHENS**						
2017	Thirty eight lichen acetone extracts of nine different families (mainly Parmeliaceae and Cladoniaceae)	*C. albicans* ATCC 3153		Seven extracts displayed anti-maturation effect. Among them, *Evernia prunastri* and *Ramalina fastigiata* were the most promising lichens (IC_50_ < 4 µg/mL)	Seven extracts showed antibiofilm capacity. Among them, *E.prunastri*, *Cladonia uncialis* (*R. fastigiata* and *Xanthoparmelia conspersa* (showed IC_50_ values <10 µg/mL	[143]
**MICROALGAE AND CYANOBACTERIA**						
2019	675 hexane, ethyl acetate and methanol extracts obtained from 225 microalgae and cyanobacteria	*C. albicans* and *C. parapsilopsis* (voucher N°s not stated)		*C. albicans* and *C. parapsilopsis* biofilm formation was inhibited by 308 extracts. *C. albicans* biofilms were particularly sensitive to extracts from Cryptophyta, Euglenophyta, and Glaucophyta (three completely unrelated lineages), with MBIC_50_ = 8 µg/mL. Instead, *Rhodophyta* spp. showed activity against *C. parapsilopsis* with MBIC_50_ = 64 µg/mL		[144]

ATCC: American type culture collection; CFU: colony forming units; EtOH: ethanol; IC_50_: concentration that inhibits 50% growth; MBIC: minimum biofilm inhibitory concentration; MIC: minimum inhibitory concentration.

**Table 4:** More relevant results of natural compounds showing a capacity for inhibiting fungal biofilms.

Year	Type of Compound/ Natural Source	Structure and Name		Strains	In Vitro Type of Studies				Reference
					Inhibition of Cell Adhesion or Hyphal Formation	Biofilm Formation	Eradication of Mature Biofilms	Studies of Mechanisms of Action	
2017	Formyl-phloroglucinol meroterpenoid Source *Eucalyptus* spp. and *Psidium* spp.	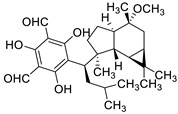 eucarobustol E (EE)		*C. albicans* SC5314 and ATCC 24433; 10 Fluconazole-resistant *C. albicans* and 8 Fluconazole-susceptible *C. albicans*	EE inhibited *C. albicans* yeast-to-hyphal transition in both liquid and solid hypha-inducing media	EE inhibited 60 and 73% biofilm formation at 16 and 32 µg/mL, respectively and 100% at >32 µg/mL	EE eradicated mature biofilm at 128 µg/mL	At 8 and 16 µg/ml EE reduced by 9.2% and 65.3% the ergosterol production and increased by 3.95- and 5.43-fold the farnesol production, respectively	[145]
2017	Isoquinoline alkaloid Source Not informed	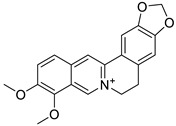 berberine (BBR)		*C. albicans* SC5314; *C. parapsilosis* ATCC 22019; *C. glabrata* ATCC 15126; *C. tropicalis* ATCC 750 and other *C tropicalis* strains (2203, 2317, 2006, 2718, 333, 087, 20026)	BBR inhibited *Candida* biofilm formation with MBIC values of 64–256 µg/mL				[146]
2017	3-3’-Neolignan Source *Magnolia officinalis*	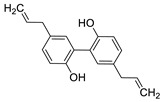 magnolol. (2-(2-hydroxy-5-prop-2-enylphenyl)-4-prop-2-enylphenol)		*C. dubliniensis* CDC 27897; *C. albicans* CDC 27907 and ATCC 24433; *C. glabrata* CDC 28621			Magnolol inhibited 35.6%–69.5% preformed biofilms of the three fungi at 32 µg/mL		[147]
2017	5 terpenes and one phenylpropanoid. Sources. EOs from *Origanum vulgare*, *Mentha piperita*, *Thymus vulgaris Cinamomum verum Cymbopogon citratus* and *Syzygium aromaticum*	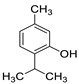	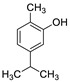	*C. neoformans* NCIM 3541 and *C. laurentii* NCIM 3373		MBIC_80_ against *C. neoformans* and *C. laurentii*: for thymol: 32 and 16 µg/mL; for carvacrol, 64 and 32 µg/mL and for citral, 128 and 64 µg/mL, respectively.	MBEC_80_ of thymol and carvacrol against *C. neoformans* were 128 and 256 µg/mL, respectively, and against *C. laurentii* 64 and 128 µg/mL, respectively. MBEC_80_ for citral was 256 µg/mL for both fungi	The compounds reduced EPM, cellular density and altered the surface morphology of biofilm cells	[148]
		thymol	carvacrol						
		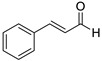 cinnamaldehyde							
		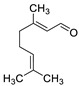 citral	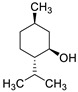 menthol						
		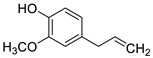 eugenol							
2017	Eudesmane sesquiterpene Source *Tritomaria quinquedentata*	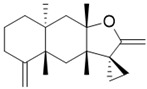 *ent*-isoalantolactone (*ent*-iLL)		*C. albicans* DSY654. Genotype: *Dcdr1::hisG/Dcdr1::hisG/Dcdr2::hisG-URA3-hisG/* *Dcdr2::hisG*	*Ent*-iLL inhibited the yeast-to-hyphal switch at 4 or 8 µg/mL in agar plate tests or liquid medium, respectively			*Ent*-iLL reduced ergosterol content by inhibiting Erg11 and Erg6	[151]
2017	β-diketone diphenol Source *Curcuma longa* (Zingiberaceae)	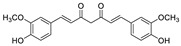 curcumin (Cur)		*C. albicans* SC5314	Cur at 50 µg/mL reduced the capacity of *C. albicans* to attach to polymethyl methacrylate (PMMA) denture base material	The MBIC_80_ for Cur was ≥200 μg/mL for sessile cells		Cur at 50 μg/mL down-regulated the adhesin ALS3, with minimal impact on ALS1. The clustered aggregative and flocculation genes *AAF1*, *EAP1*, and *ALS5* transcripts were up-regulated	[152]
2018	Phenols with a variable length in the lateral chain (6 or 8 carbons) bearing a keto substituent and a *β*-OH (gingerols) or a Δ-5 double bond (6- shogaol) Source: *Zingiber officinale*	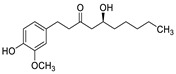 6-gingerol (6-G) 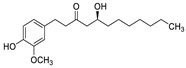 8-gingerol (8-G) 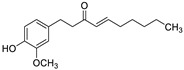 6-shogaol (6-S)		Fluconazole-resistant *C. albicans* DAY185	6-S at 10 µg/mL was more effective in suppressing hyphal formation than 6 g at 50 µg/mL	6-S at 10, 50, and 100 µg/mL inhibited 85, 94, and 94% biofilm formation, respectively. 6 g and 8 g at 50 µg/mL inhibited by 88 and 80%, respectively the biofilm formation		6 g and 6-S significantly altered the expressions of some hypha-specific (*HWP1* and *ECE1*), biofilm-related (*HWP1* and *RTA3*) and multidrug transporter (CDR1 and CDR2) related genes. 80% of *C. elegans* infected with *C. albicans* survived in the presence of both compounds at 50 μg/mL	[153]
2019	Monoterpenes Source *Origanum vulgare* *Cinamomum verum* *Cymbopogon citratus * *Mentha piperita* and *Thymus vulgaris*	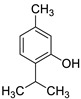 thymol	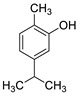 carvacrol	*C. neoformans* NCIM 3541 (equivalent to ATCC 32045)		The three compounds inhibit biofilm formation and eradicate mature biofilms by the following mechanisms: (i) ergosterol biosynthesis inhibition and selectively interaction via ergosterol binding, (ii) disruption of the biofilm cell surface with reduction in cell height, alterations in the fatty acid profile which attenuate the cell membrane fluidity with enhanced permeability, resulting in pore formation and efflux of the K+/intracellular content, (v) mitochondrial depolarization caused higher levels of ROS. Then, the oxidative stress caused a significant decline in the amount of EPM and capsule sugars (mannose, xylose, and glucuronic acid), leading to a reduced capsule size and an overall negative charge on the cell surface			[150]
		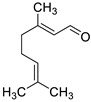 citral							
2019	1,4-naphtoquinone derivative Source (*Lithospermum* *erythrorhizon*)	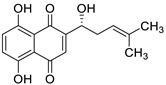 shikonin (SK)		*C. albicans* SC5314 and 10 clinical isolates from Changhai Hospital of Shanghai, China.	The filamentation in Lee’s media was completely inhibited by 0.5 µg/mL of SK	SK at 4 µg/mL inhibited biofilm formation by 65.4%, while the biofilm growth was almost totally inhibited when exposed to 32 µg/mL of SK	SK at 32 µg/mL destroyed mature biofilms by 92.8%	The expression of genes involved in hyphae formation and adhesion, *ECE1, HWP1, EFG1, CPH1,RAS1, ALS1, ALS3* and *CSH1* were downregulated while *TUP1, NRG1*, and *BCR1* were upregulated	[154]
2020	Phenolic aldehyde Source Many plants including *Melia azedarach*	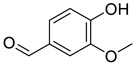 vanillin (Van)		*C. albicans* SC5314, MRC10 (Δicl1) and MRC11. Consult the genotypes in the original reference	With Van at 62.5 µg/mL, *C. albicans* were unable to express filaments and presented a normal morphology with few or no adherence to buccal epithelial cells.	Adherence to polystyrene surface and biofilm formation were reduced by 49%	Mature biofilm eradication (52%) was observed	*C. albicans* biofilms were absent or negligible in *C. elegans* worms treated with 125 µg/mL of Van	[155]
2020	Sesquiterpene alcohol Source Dimorphic fungus *C. albicans*	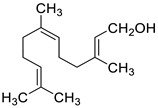 farnesol (FS)		*Fusarium keratinoplaticum* ATCC 36031	FS prevented the adhesion of conidia and filamentation for biofilm formation	FS reduced the number of viable cells and the total biofilm biomass. The metabolic activity was only reduced at 500 μM. At 700 μM, FS completely prevented the biofilm formation	FS was able to modulate preformed biofilms, decreasing significantly the number of viable cells, in particular at >600 μM		[156]
2020	Labdane diterpenoid Source liverwort *Heteroscyphus* *coalitus*	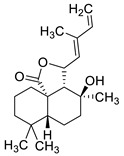 heteroscyphin D (HSc-D)		CDR1 and CDR2 efflux pumps deficient strain *C. albicans* DSY654 and the wild type *C. albicans* S5314	HSc-D restricted the formation of hyphae at 4 μg/ml, but showed no activity against SC5314. HSc-D decreased the adherent *C. albicans* cells on A549 cancer cell monolayers from 1 µg/mL	HSc-D completely prevented biofilm formation at ≥8μg/mL		HSc-D decreased the transcriptional levels of the genes *ALS3*, *HWP1*, and *ECE1* encoding adhesins and affected the Ras1-cAMP-Efg1 pathway, *NRG1* and *UME6* to retard the yeast-to-hyphal transition.	[157]
2020	Artemisinin sesquiterpene lactone Scopoletin: coumarin derivative Sources Ar: *Artemisiaannua* Sc: from several plants including *A. annua*	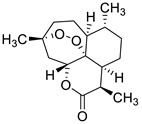 artemisinin (Ar) 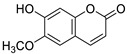 scopoletin (Sc)		*C. albicans* 1372; *C. dubliniensis* 1470, *C. tropicalis* 1368, *C. krusei* 779, *C. glabrata* 1374 and *C. guilliermondii* 808		FS and Sc reduced biofilm biomass and metabolic activity and led to non-viable cells	Ar was more effective in disrupting the preformed EPM- structure and in killing the sessile cells as compared to Sc at their respective MBEC_10_. In *C. albicans, C. dubliniensis* and *C. glabrata*	Ar and Sc promoted the accumulation of intracellular ROS by increasing oxidative stress at their respective MBEC_10_	[158]
2020	Scopoletin: coumarin derivative Source *Mitracarpus frigidus*.	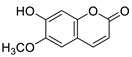 scopoletin (Sc)		Fluconazole, itraconazol and amphothericin--resistant *C. tropicalis* ATCC 28707		At 4× MIC (200 µg/mL), Sc produced a great reduction of the area occupied by biofilms on the surface of coverslips	At its MIC (50 µg/mL), Sc reduced preformed *Candida* biofilms		[159]
2020	Drimane sesquiterpene dialdehydes Source *Warburgia ugandensis*	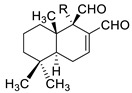 (+)-warburganal R=OH (-)-polygodial R=H		*C. albicans* SC5314; *C. glabrata* ATCC 2001 and *C. glabrata* BG2		Warburganal: MBIC_50_ = 4.5 and ~50 µg/ml against *C. albicans* and *C. glabrata*, respectively. Polygodial: MBIC_50_ = ~10 and and ~50 µg/mL, respectively	Warburganal and polygodial: MBEC_50_ = ~ 16 µg/ml against *C. albicans* but did not eradicate the C. *glabrata* biofilm		[160]
2020	Dammarane-type glycosides (gypenosides) Source *Gynostemma pentaphyllum*	No description of the compounds tested 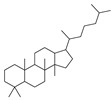 dammarane skeleton		Flu-resistant *C. albicans* CA10 and CA16		Gypenosides showed MBIC_80_ > 128 µg/mL			[161]
2021	Polyciclic compound with a lipophilic side chain Source Sea squirt microbiome constituent *Micromonospora* sp.	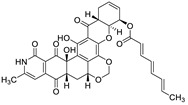 turbinmicin (TBM)		*C. albicans* SN250, *C. tropicalis* 98-234, *C. glabrata* 4720, *C. auris* B11220 and *A. fumigatus* 293		At 2 and 4 µg/mL, TBM reduced the biofilms by 50%	TBM inhibited the biofilm extracelular vesicle (EV) production and, thus, eliminated the EPM assembly	Ten μg/mL of TBM eliminated *C. albicans C. tropicalis, C. glabrata, C. auris* and *A. fumigatus*, biofilms from catheters	[162]
2021	Organosulfur compound. Diallyl thiosulfinate Source (*Allium sativum*)	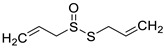 allicin		*C. albicans* ATCC 14053			Allicin eradicated 50% *C. albicans* biofilms at sub-MIC = 4 µg/mL		[164]
2021	Terpenphenol __________ Source *Cannabis sativa*	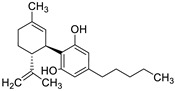 cannabidiol (CBD)		*C. albicans* SC5314 *C. albicans* SC5314 carrying the green fluorescent protein (GFP) reporter gene (*C. albicans*–GFP)		CBD at 25 µg/mL caused a pronounced inhibitory biofilm formation effect. MBIC_90 =_ 100 µg/mL	At 1.56 and 3.12 µg/mL, mature biofilm decreased 28% and 44%, respectively.	CBD showed a multitarget mode of action with up- regulation of yeast-associated genes and downregulation of hyphae-specific genes. *C. albicans* virulence genes decreased. CBD increases ROS production, reduces the intracellular ATP levels, modifies the cell wall, and increases the plasma membrane permeability	[165]
2021	Monoterpenes Sources EO from many species of genera such as *Cinnamomum*, *Eucalyptus*, *Artemisia*, *Salvia* and *Thuja*	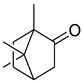 camphor 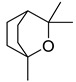 eucalyptol		*C. albicans* 475/15, *C. albicans* 503/15, *C. albicans* ATCC 1023,; *C. krusei* H1/16; *C. tropicalis* ATCC 750 and *C. parapsilosis* ATCC 22019	Camphor at 0.125 mg/mL and eucalyptol at 23 mg/mL induced a notable reduction in the number of hyphal cells in *C. albicans* 475/15	Camphor and eucalyptol inhibited *C. albicans,* *C. tropicalis,* *C. parapsilosis* and *C. krusei* biofilm biomass		Camphor at 0.125 mg/mL reduced ROS by 52% while eucalyptol was inactive	[166]

ATCC: American type cultuire collection (Manassas, VA, USA); CDC: comprehensive dental care number, provided by Kuwait University Dental Clinic (KUDC); EOs: essential oils; EPM: extracellular polymeric matrix; MBEC: minimum effective concentration; MBIC: minimum biofilm inhibitory concentration; NCIM: National collection of industrial microorganisms, Pune; ROS: reactive oxygen species; SC: obtained from professor D. Sanglard, Centre Hospitalier Universitaire Vaudois, Lausanne, Switzerland; SMIC: sessile minimum inhibitory concentration. Other culture collection acronyms can be found in the respective reference.

**Table 5:** Nanososystems containing natural products.

Nanosystems formed with essential oils (EOs)								
**Year**	**EOs Included in Nanosystems**	**Type of Nano System**	**Fungal Strains**	**Inhibition of Cell Adhesión or Hyphal Formation**	**Biofilm Formation**	**Eradication of Mature Biofilms**	**Mechanisms of Action or Genes Expression**	**Reference**
2017	*Eucalyptus globulus* EO	Nanoemulsions (NE) __________ **Composition** Oil phase: 5% EO and 2% sorbitan monooleate Aqueous phase: 2% Tween 80 and 25 mL of water	*C. albicans* ATCC 14053; *C. tropicalis* ATCC 66029; *C. glabrata* ATCC 66032		(EO-NE) at 22.5 mg/mL reduced *C. albicans, C. tropicalis* and *C. glabrata* biofilm formation			[169]
2017	*Melaleuca alternifolia* EO (tea tree oil, TTO)	Nanoparticles (NP) __________ **Composition** Proprietary method from Inventiva^®^ (Porto Alegre, Brazil)), based on high pressure homogenization. 7.5% (*w*/*v*) of TTO, cetyl palmitate as the solid lipid and Tween 80 as the surfactant	*C. albicans* ATCC 14053; *C. glabrata* ATCC 66032; *C. parapsilopsis* ATCC 220190; *C. tropicalis* ATCC 66029; *C. membranaefaciens* ATCC 2013770		TTO-NP at 15.6% decreased the biofilm formation of all strains tested. The antibiofilm activity was higher in *C. glabrata*		TTO-NP decreased EPM and protein content in biofilms, and TTO-NPs inhibited the enzyme succinate dehydrogenase	[171]
Nanosystems formed with natural compounds								
**Year**	**Type of Compound** **Name and Structure** **Natural Source**	**Nanosystem**	**Fungal Biofilms spp.**	**Inhibition of Cell Adhesion or Hyphal Formation**	**Biofilm Formation**	**Eradication of Mature Biofilms**	**Studies of Mechanisms of Action**	**Reference**
2020	β-Diketone diphenol 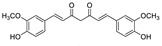 curcumin (Cur) Source *Curcuma longa*	Positively charged chitosan nanoparticles (CSNP) Cur was loaded on CSNP, forming CSNP-Cur	*C. albicans* DAY185		CSNP-Cur at 200 μg/mL inhibited almost completely the *C. albicans* biofilm formation	CSNP-Cur at 400 μg/mL eradicated the preformed *C. albicans* biofilms		[172]
2020	1,4-Naphtoquinone derivative 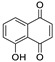 juglone (JU) Source *Juglans regia*	Nanoemulsions JU was loaded on poly (*D*,*L*-lactic-co-glycolic acid) (PLGA) forming PLGA-JU	*C. albicans,* non-specified voucher	PLGA-JU reduced the cell adhesion at 1.25 and 0.625 mg/mL	PLGA-JU at 1.25 and 0.625 mg/mL inhibited by 100% the *C. albicans* biofilm formation, being more effective than free JU and Fluconazole	PLGA-JU at doses equivalent to 1.25 and 0.625 mg/mL of JU completely inhibited pre-established *C. albicans* biofilms	PLGA-JU caused membrane depolarization of biofilm cells	[173]
2021	β-Diketone diphenol compound 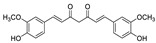 curcumin (Cur) Source *Curcuma longa*	Cur-SL Nanocomplex formed with the surfactant sophorolipid (SL) Cur was loaded to the nanoparticle prepared with SL forming Cur-SL	*C. albicans* DAY185	CU-SL at sub-inhibitory concentrations of 9.37 μg/mL significantly suppressed fungal adhesion	CU-SL at 9.37 μg/mL inhibited biofilm development and maturation		Four major transcriptional genes that promote biofilm formation, *ROB1*, *EFG 1, TEC1, BRG 1* and *NDT80* were down-regulated, as well as the adhesin genes *ALS1, SAP8* and *EAP1,* the hyphal regulatory genes *SAP4, HWP1 RAS1* and *HYR1* and *ERG11*	[174]

ATCC: American type cultuire collection (Manassas, VA, USA); EO: essential oil; NE: nanoemulsions; NP: nanoparticles. Other culture collection acronyms can be found in the respective reference.
